# The change of gait analysis on applying metatarsal-bar used 3D motion analysis

**DOI:** 10.1186/1757-1146-7-S1-A99

**Published:** 2014-04-08

**Authors:** Se won Yoon, Jeong woo Lee, Soo ji Park, Woong sik Park, Moon jeong Kim

**Affiliations:** 1 Department of physical therapy, Kwangju women’s university, Kwangju, 506-713, Korea; 2 Department of physical therapy, Graduate school, Kwangju women’s university, Kwangju, 506-713, Korea; 3 Department of occupational therapy, Kwangju women’s university, Kwangju, 506-713, Korea; 4 Department of physical therapy, Shinhwa rehabilitation clinic, Busan, Korea

## Introduction

Enough dorsiflexion angle of the first metatarsophalangeal joint is needed to ensure thrust and stability [1], and if the range of motion of the first metatarsophalangeal joint decreases, normal foot function during gait is under severe restriction [2]. Wearing shoes with high heels during gait makes weight leaning forward and wearing them for long may cause deformation of hallux valgus [3,4]. General therapeutic concepts of foot diseases focus on reducing or eliminating plantar tissue stress. Recently, studies on treatment to solve these problems have been conducted, but there have been a few studies on orthotic devices such as metatarsal pad, dome and wedge [5].

Therefore, this study applied bar metatars ophalangeal area of normal persons and examined if any change occurs of spatio-temporal indices and kinematic parameters by using 3D motion analysis system.

## Method

This study selected 40 female university students in their twenties and conducted the experiment with them before and after applying metatarsal bar. Spatio-temporal indices including stance phase, swing phase, double phase, cadence, stride time, stance phase time, swing phase time, double stance phase time, step length, stride length, velocity, stride velocity, and swing phase velocity were measured through 3D motion analysis system and kinematic parameters such as pelvic tilt angle, hip joint flexion-extension angle, knee joint flexion-extension angle, foot progression angle, and ankle joint flexion-extension angle were also measured through 3D motion analysis system.

After attaching metatarsal bar to the subjects on bare foot, they had enough practice in laboratory to make them accustomed to gait. Before experiment, calibration was conducted, gait space was measured and then subsequent experiment was carried out.

Marker was at attached to joint and photographing was made at the condition that reflective materials except marker were eliminated within the camera view. Marker was at attached to sacrum (1), anterior superior iliac spine (left, right), greater trochanter (left, right), femur ½ location (left, right), lateral epicondyle of femur (left, right), fibular head (left, right), tibia ½ location (left, right), lateral malleolus (left, right), 5th metatarsal head (left, right), and heel (left, right). Marker should be attached to be seen in a straight line from side to measure the accurate angle and the subjects were made to stand in the middle of gait path and look at the front.

The subjects are made to stand at the starting point of the path to measure the dynamic gait and their gaits were measured after comfortable walking by measurer’s instruction. The subjects wore shorts to prevent the sway of marker during gait and preferred walking velocity was used as gait velocity.

## Result

As a result of analyzing gait, knee joint angle showed significant difference and remaining variables showed no significant difference.

## Conclusion

This study was showed significantly decreased that change of knee joint angle on applying metatarsal-bar. It was the same change when wearing high heel appears that general knee joint decreased. We think that applying metatarsal-bar wasn’t influence on change of knee joint angle. Also gait parameter on applying metatarsal-bar showed no significantly difference, we suggest that metatarsal-bar is helpful to foot diseases patients.
